# O.1.3-2 Development and implementation of physical activity policies at national and subnational levels: a pilot study in Latin American countries

**DOI:** 10.1093/eurpub/ckad133.099

**Published:** 2023-09-11

**Authors:** Juliana Mejía Grueso, Michael Pratt, Eugen Resendiz, Gloria Isabel Niño Cruz, Nubia Yaneth Ruiz Gómez, Rafael Alexander Leandro Gómez, Inés Revuelta Sánchez, Gerardo Alonso Araya Vargas, Angélica María Ochoa Avilés, Alejandra Jáuregui, Deborah Salvo, Andrea Ramírez Varela

**Affiliations:** School of Medicine, Universidad de los Andes, Bogotá, Colombia; Herbert Wertheim School of Public Health and Human Longevity Science, University of California San Diego, USA; j.mejia11@uniandes.edu.co; Prevention Research Center, Brown School, Washington University in St. Louis, St. Louis, Missouri, USA; School of Medicine, Universidad de los Andes, Bogotá, Colombia; School of Physiotherapy, Universidad Industrial de Santander, Bucaramanga, Colombia; Physical Activity Internal Group, Ministerio del Deporte, Colombia; Physical Activity Internal Group, Ministerio del Deporte, Colombia; School of Human Movement Sciences and Quality of Life, Universidad Nacional, Costa Rica; School of Human Movement Sciences and Quality of Life, Universidad Nacional, Costa Rica; School of Physical Education and Sports, Universidad de Costa Rica, Costa Rica; Department of Bioscience, Universidad de Cuenca, Ecuador; Department of Physical Activity and Healthy Lifestyles, Center for Nutrition and Health Research, National Institute of Public Health of Mexico, Cuernavaca, Mexico; Department of Kinesiology and Health Education, The University of Texas at Austin, Austin, USA; School of Medicine, Universidad de los Andes, Bogotá, Colombia

## Abstract

**Purpose:**

National physical activity (PA) policy processes are only beginning to be documented, and the interaction between the actors involved in these processes remains understudied. This study was designed to gain further insights into the policy process (agenda setting, policy formulation, adoption, implementation, evaluation) and the interaction between national and subnational levels.

**Methods:**

We conducted a pilot study to assess the feasibility and scalability of an instrument to assess the PA policy process as part of the work of the Global Physical Activity Observatory (GoPA!). The instrument was tested in Colombia, Costa Rica, Ecuador, and Mexico, where pilot data were collected in matched pairs of the capital plus one non-capital city among national and subnational policy makers (n = 24), as identified by the GoPA! Country Contacts. Descriptive statistics were calculated to assess perceptions on the influences and interactions across the two levels with respect to PA policy development, implementation, and evaluation.

**Results:**

Twenty-two (response rate=85.7%) key informants provided data, mostly from the health sector (54.5%), followed by sports (27.3%), education (9.1%), and transport (9.1%) government institutions. Most participants reported that their countries have a current PA policy embedded within a noncommunicable diseases prevention plan (50.0%), other plan (41.7%), or a plan to prevent, manage, or control obesity (8.3%). Respondents at the subnational level rated PA promotion as central (75.0%), while at the national level the role was important but not central (75.0%). Across the five stages of the policy process, national and subnational policy makers reported low-to-little involvement in the other level’s PA policy processes.

**Conclusions:**

This pilot study demonstrated the feasibility of collecting national and subnational PA policy data across countries with the active collaboration of the GoPA! network Country Contacts. Also, we successfully identified the degree of interaction between government levels throughout the PA policy process. Initial results suggest that engagement between national and subnational levels is suboptimal.

**Funding:**

Universidad de los Andes, Bogotá, Colombia; University of California San Diego, USA.

